# Gender Differences in Health Related Quality of Life among People Living with HIV on Highly Active Antiretroviral Therapy in Mekelle Town, Northern Ethiopia

**DOI:** 10.1155/2015/516369

**Published:** 2015-01-06

**Authors:** Amanuel Tesfay, Abebe Gebremariam, Mulusew Gerbaba, Hailay Abrha

**Affiliations:** ^1^Department of Population and Family Health, College of Public Health and Medical Sciences, Jimma University, P.O. Box 2080, Jimma, Ethiopia; ^2^Department of Epidemiology, College of Public Health and Medical Sciences, Jimma University, P.O. Box 2080, Jimma, Ethiopia

## Abstract

*Background*. Health related quality of life (HRQOL) is an important outcome measure for highly active antiretroviral treatment program. In Ethiopia, studies revealed that there are improved qualities of life among adults living with the viruses taking antiretroviral therapy but there is no explicit data showing gender differences in health related quality of life. *Aim*. To assess gender differences in HRQOL and its associated factors among people living with HIV and on highly active antiretroviral therapy in public health institutions of Mekelle town, Northern Ethiopia. *Methods*. A comparative cross-sectional study was conducted among 494 adult people living with HIV taking ART services. Quality of life was measured using WHOQOL-HIV BREF. *Result*. There was a statistically significant gender difference (*P* < 0.05) in HRQOL among PLHIV on HAART. Females had low score in all HRQOL domains. High perceived stigma was strongly associated with poor psychological quality of domain among both female and male groups with [AOR = 2.89(1.69,4.96)] and [AOR = 2.5(1.4,4.4)], respectively. *Conclusion*. There was statistically significant gender difference in all quality of life domains. Public health interventions to improve HRQOL of PLHIV should take in to account the physical, psychological, social, environmental, and spiritual health of PLHIV during treatment, care, and support.

## 1. Introduction

HIV/AIDS remains one of the key challenges for the overall development of Ethiopia, as it has led to a seven-year decrease in life expectancy and a greatly reduced workforce [1]. In 2010 it was estimated that there are 1.2 million PLHIV, with an adult HIV prevalence of 2.4% (7.7% urban and 0.9% rural) and more prevalence in females (2.9%) than in males (1.9%). A total of 397,818 people living with HIV (PLHIV) were estimated to be in need of antiretroviral treatment (ART) in 2010 [2]. As some studies indicated, HIV/AIDS has changed individual's lifestyles and quality of life. Empirical evidence shows that as the HIV disease progresses, quality of life deteriorates [3, 4]. PLHIV face physiological, physical, psychological, and sociocultural problems that are caused by many factors such as symptoms of the virus, side effects of the antiretroviral treatment, and opportunistic infections [5]. HIV/AIDS has multidimensional consequences: personal suffering such as discomfort associated with the disease's progression, the social impact of the diagnosis, the emotional consequences of dealing with the diagnosis, and related stigma.

Hence it interferes with day-to-day functioning and affects both personal relationships decision making and economic hardships. The importance of finding ways of mitigating these consequences of HIV/AIDS makes quality of life in PLHIV a salient issue for health care [6].

Government of Ethiopia introduced its free ART program by early 2005, with the goal of reducing HIV-related morbidity and mortality, improving the quality of life of people living with HIV and mitigating some of the impact of the epidemic [7, 8]. Given the longevity achievable with the current prophylactic and therapeutic strategies for PLHIV quality of life has emerged as a significant measure of health outcome and quality of life enhancement as an important goal [9]. The concept of quality of life can be traced back to 1947 in the World Health Organization's (WHO) definition of health [10].

Although there is still no agreed upon definition, there is agreement that quality of life is a multidimensional construct defined in terms of an individual's subjective experiences and a construct that cannot be generalized across cultures [3] However, World Health Organization (WHO) defines quality of life as an individual's perception of their position in life in the context of culture and value systems in which they live and in relation to their goals, expectations, standards, and concerns [11, 12]. Even if the national strategic objectives of accelerated access to prevention, care, and treatment of Government of Ethiopia are to create care and support for people living with HIV to improve their quality of life, adequate attention had not been given to measuring and monitoring the qualitative outcome of HAART through health related quality of life measurements. Therefore there is need to assess the quality of life of both men and women to determine if there are important differences relevant to health care and interventions. Moreover, to the investigators' knowledge there is no explicit data that has examined gender differences in quality of life in Ethiopia in general and in the study area in particular using WHOQOL-BREF instruments. Hence, the aim of this study was to assess gender differences in quality of life among adult PLHIV on antiretroviral therapy.

## 2. Methods and Participants

The study was conducted in Mekelle town, Tigray Region, from February 15 to April 15 2012. Mekelle town is located 776 Kms north of the capital city Addis Ababa. A facility based comparative cross-sectional study was employed among randomly selected adult PLHIV on HAART who have regular follow-up that were selected from five health institutions found in the town, namely, Mekelle Hospital, Ayder Hospital, Mekelle Health Center, Kasech Health Center, and Semien Health Center. Those in age groups above or equal to 15 years have been on treatment for more than 3 months and with available CD4 count were included in the sample. As the study focused on measuring health related quality of life in female and male groups, differences as little as 3–5 points are considered to be clinically important differences [13]. Sample size for the two groups was determined by Open Epi software version 2.3 considering the following parameters: power 80%, 95% CI, a 1 : 1 ratio for male versus female PLHIV on HAART, and mean 81.2 (SD ± 14.19) for males and mean 77.1 (SD ± 17.40) for females [14] to detect a difference of 4.15. Thus, considering a 10% nonresponse rate, the final sample size becomes 506 (253 males and 253 females) and this was allocated proportionally. List of PLHIV who have an appointment during the study period (two months) was used as a sampling frame. Then the study participants were selected using computer generated random numbers from each ART site using their unique ART numbers. Refusals were considered as nonresponse.

Five senior nurses/data clerks one from each study site extract the data on clinical characteristics of PLHIV on HAART according to the selection criteria and five trained peer to peer counselors interviewed the participants on the sociodemographic, psychosocial and quality of life information of study subjects while the investigators and one health officer supervised to assure the quality. The questionnaire was adapted to the study setting context and translated into Tigrigna (local language). The translation of the English version of the questionnaire into Tigrigna (local) language was done using standard methods which consist of three phases: translation, back-translation, and harmonization.

Two independent forward translations into Tigrigna were carried out by native Tigrigna speakers fluent in English. After a critical inspection by the principal investigator and mental health specialist, a first version of the translation was agreed upon. Then it was back-translated into English by three independent bilingual translators of Tigrigna speakers English language professionals. All items which show differences between the original and the back-translated version were thoroughly discussed with the principal investigator and the translators. Finally, it was pretested on 5% of sample size for three days using Tigrigna translated and locally adapted WHO QOL-HIV BREF interview questionnaire in PLHIV on HAART who have follow-up other than the study facilities which have similar characteristics with the study subjects.

Health related quality of life was measured using the interviewer administered World Health Organization's Quality of Life HIV short form instrument (WHOQOL-HIV BREF) [15].

The WHOQOL-HIV BREF instrument produces six domain scores and contains 31 items. For each item there is a five-point Likert scale where 1 indicates low or negative perceptions and 5 high or positive perceptions. These items contain six domains: physical health (4 items), psychological well-being (5 items), social relationship (4 items), environmental health (8 items), level of independence (4 items), and spiritual health (4 items). There are two items that examine general quality of life: question 1 asks about an individual's overall perception of quality of life and question 2 asks about an individual's overall perception of his or her health. The physical health domain contained information on presence of pain, energy, and sleep. The psychological domain consisted of negative and positive feelings, self-esteem, and thinking. The social domain covered social support, personal relationship, and sexual activity. Mobility, work capacity, and activities were included in the level of independence. Financial issues, home and physical safety and security, and participation in leisure activities were included under the environment domain. The spirituality domain did contain questions about death and dying, forgiveness and blame, and concern about the future. The suggested reference time frame of QOL experienced within two weeks was used in this study [16].

Information on demographic, behavioral, and psychosocial variables such as perceived stigma was also collected through face to face interview using pretested interviewer administered questionnaires. Perceived stigma was measured using adopted instrument that has been used in local context for the same population (PLHIV on HAART) [17, 18]. The stigma items consist of four-point Likert scale (strongly disagree, disagree, agree, and strongly agree) questions. Questions were asked about perceived isolation, shame, guilt, and disclosure of the HIV status. The stigma score ranges from 23 to 92. A person was said to be in high perceived stigma when an individual scored above or equal to the mean stigma score and low perceived stigma when an individual scored below the mean stigma score.

## 3. Operational Definitions

### 3.1. Adherence

Patients' self-report adherence was measured by asking about the number of doses missed during the past 7 days and translated quantitatively in to percentage adherence. Optimal adherence means a patient must take more than 95% of their doses (i.e., missing less than 3 doses in a month).

### 3.2. Psychosocial Support from Family Members

It is support for the PLHIV from family members or friends in terms of psychological, financial, or adherence support.

### 3.3. Psychosocial Support outside Family Members

It is support for the PLHIV outside family members like governmental or nongovernmental organizations, religious based organizations. and community based organizations in terms of psychological, financial, material, or spiritual support.

Data on CD4 count, WHO staging, reported side effects, drug adherence, and functional status of participants were extracted from HIV CARE/ART registries.

Data were analyzed using the SPSS version 16.0 software. First the descriptive statistics were calculated for sociodemographic characteristics, behavioral, psychosocial, and clinical variables in terms of mean, median, standard deviations, and range values for numerical data as opposed to percentage and frequency tables for categorical data.

Domain scores in the WHOQOL-HIV were scaled in positive direction with higher score denoting good quality of life. Negative questions like pain and discomfort were recorded so that higher scores reflected better QOL. Mean scores of the items within each domain were used to calculate the domain score. Mean scores were then multiplied by 4 in order to make domain score comparable with the scores used in WHOQOL-100. Independent sample *t*-test was used to compare means between groups for domains that fulfill assumption. By taking the mean of each domain as a cutoff point, QOL was dichotomized as poor or good. Individuals who scored below the mean were classified as having poor quality of life on each of the six domains.

To assess predictors of QOL (poor versus good), first univariate analysis was employed and then variables that show statistically significant association with each of the six WHOQOL domains QOL in the univariate analysis (*P* < 0.2) were entered into a stepwise multiple variable logistic regression model separately for each gender. A significance level was set at *P* < 0.05. Finally, goodness of fit of the final model was checked using Hosmer and Lemeshow statistic.

Ethical clearance was obtained from the health research and postgraduate coordinating office of College of Public Health and Medical Sciences of Jimma University. Permission letter was obtained from study facilities and all information was confidentially used only for research purpose.

## 4. Result

### 4.1. Sociodemographic and Psychosocial Variables and Clinical Parameters

A total of 250 females and 244 males responded to the questionnaires making a response rate of 97.6%. Most of the respondents, 332 (67.2%), were attending their follow-up services at Mekelle Hospital followed by 56 (11.3%) at Mekelle Health Center. Their mean age was 35.46 (SD ± 8.03) years for females and 39.75 (SD ± 7.85) years for males. One hundred sixty-nine (67.6%) females and 214 (87.7%) males were literate. One hundred seventy-eight (71.2%) females and 232 (95.1%) males reported to have some form of employment. Two hundred thirty-four (93.6%) females and 231 (94.7%) males were Orthodox Christians (Table 1).

One hundred seventy-one (68,4%) females and 156 (63.9%) males reported they did not receive any psychosocial support from family members/friends; in contrast 179 (71.6%) females and 164 (67.2%) males reported they get psychosocial support outside of their family. members or friends such as governmental and nongovernmental, and religious organizations. Median CD4 count at start of ART was 163 (IQR = 95–240) for females and 137 (IQR = 66–215.5) for males, respectively. And most recent CD4 cell counts were 366 (IQR = 250–523) for females and 296 (IQR = 205–441) cells/mm^3^ for males (Table 1).

### 4.2. Validity and Reliability of Tigrigna WHOQOL-BREF Version

The internal consistency (Cronbach's *α* coefficient) of the Tigrigna WHOQOL-BREF tool was moderate ranging from 0.53 to 0.65 in the HRQOL domains and 0.87 for the total items. It ranges between 0.86 and 0.87 for all the items if each item was deleted.

The interdomain correlation showed that there was statistically significant association between domains. Weak correlation was observed between spiritual and environmental domains (*r* = 0.14, *P* < 0.01), and strong correlation between level of independence and physical health (*r* = 0.67, *P* = 0.00).

### 4.3. Gender Difference in Health Related Quality of Life Domains

The overall mean ± SD quality of life perception was 3.96 ± 0.91 and 4.11 ± 0.85 for females and males, respectively. Similarly the mean ± SD score for general health perception score was 4.08 ± 3.52 and 4.27 ± 0.79 for females and males, respectively. Perceived health related quality of life was highest in the domain spiritual/PB with mean ± SD = (17.0 ± 2.74 males, 16.41 ± 3.09 females) and the lowest social relationships domain with mean ± SD score (13.50 ± 2.71 males and 13.14 ± 2.93 females). The average scores for the females for all the six domains and two general questions were between 0.15 and 0.59 points lower than the men's score on the 4–20 scales. The difference was statistically significant for all domains and two general Questions except the social relationship domain are shown in Table 2.

### 4.4. Predictors of HRQOL

Factors strongly associated with poor HRQOL domains in men and women living with HIV on HAART in the current study were psychosocial support, perceived stigma, educational status, WHO staging, and monthly income. In female respondents those who have no psychosocial support outside family members were 2.6 times more likely to have poor physical health in relation to individuals who had psychosocial support [AOR = 2.6 (1.4,4.9)]. Similarly those illiterates were 1.8 and 3.2 times more likely to have poor physical health as compared to literates in female and male respondents, respectively. Among female respondents, those who had high perceived stigma were 2.89 times more likely to have poor psychological health as compared to those who had low perceived stigma [AOR = 2.89 (1.69, 4.96)]. Perceived stigma was also associated with poor psychological quality of domain among male PLHIV [AOR = 2.5 (1.4, 4.4)]. Educational status and family income were also significantly associated with poor social quality of life in both female and male respondents. WHO stage was significantly associated with physical and psychological domains and level of independence and environmental quality of life domains in males and with environmental domain social quality of life domain in female counterparts, respectively (*P* < 0.05).

Months on ART was significantly associated with physical, level of independence and social quality of life domains among female PLHIV (*P* < 0.05).

Perceived stigma was also significantly associated with physical and psychological quality of life and level of independence quality of life in male PLHIV (Tables 3 and 4).

## 5. Discussion

In the current study, there was statistically significant gender difference in perceived stigma among PLHIV on highly active antiretroviral treatment. This finding is similar to a study done in Thailand [19] regarding the physical, psychological, health, and overall quality of life. The study done in Vietnam [20] also found that females had significantly lower scores than males on environmental and psychological domains of HRQOL. Some investigators have suggested that gender differences in HRQOL are due to gender difference in expression of somatic complaints and psychological illness [21]. They suggest that women report poorer quality of life because their illnesses may be taken less seriously, and therefore they receive less empathy and social support than their male counterparts.

Among male PLHIV variables that predict poor physical health quality of life were illiterate, WHO clinical stage I, and those who have no psychosocial support outside family members and high perceived stigma. Male respondents in WHO clinical stage I were 85% less likely to have poor physical quality of life domain as compared to WHO clinical stage IV. A study in Brazil also reported that as the disease progresses, scores related to physical and level of independence decrease, as the clinical aggravation of the infection has an impact on the body and self-management capability [9]. The probable reason for that is that AIDS patients require greater dependence on drugs and have less work capacity due to weakness and fatal decline in health. The findings of the present study found support from the findings of the studies conducted in USA [12].

The common statistically significant predictors of poor physical health quality of life domain in both genders were being illiterate and no psychosocial support outside family members (Tables 3 and 4). This result is supported by study done in Ethiopia [20] which identifies having depression, no source of income, and no family support as determinants of poor physical health quality of life. This result may be attributable to the fact that psychosocial support could have increased personal satisfaction and positive entire effect in having self-care and good physical health perception [22].

Regarding psychological HRQOL domain in females, rural dwelling, having no psychosocial support from family members or friends, and high perceived stigma were the predictors. Similarly predictors of poor psychological health in the male counterparts were WHO stage and perceived stigma. Perceived stigma was common predictor of poor psychological health for both genders. This is comparable with research in Ethiopia [17] that identified high perceived stigma highly associated with poor psychological health.

Level of independence domain assesses persons mobility, activities of daily living, dependence on medication or treatments, and work capacity. In this study women scored lower than males in this domain are shown in Table 2.

The factors found to have significant influence on level of independence quality of life domain in multiple logistic regressions among PLHIV on HAART were residence, duration of ART, and recent side effect compliant for female PLHIV and perceived stigma, clinical staging, and monthly income for their male counterparts, respectively (Tables 3 and 4). A study in Estonia [23] supported this finding. The possible explanation for females scoring lower than males in level of independence could be that many women living with HIV are burdened by responsibility of child rising [24–26].

Reports from Thailand [19], Vietnam [20], and USA [24] assessing quality of life found no significant difference between male and female PLHIV in the domain social relationships. This is similar to our finding, even though males had slightly higher score than females which is inconsistent with study from south Africa that reported that female scored higher than males; the possible difference between the current study and results from South Africa [27] could be the high female representation (78.3%) of the study from South Africa than the current one. Amongst female respondents, no psychosocial support from family members and outside family members, age groups 15–24 and 25–34, illiterate, low monthly income, and those who were on ART for >36 months were significant predictors of poor social health quality of life domain (Table 3).

Factors associated with poor social health quality of life domain among male respondents were being single/widowed/divorced/separated, those who were on ART for >36 months, illiterate, and those who have a monthly income of average and above (≥83.75$) (Table 4). Being illiterate and low monthly income were common predictors for poor social health quality of life domain in both genders. Similarly study in Ethiopia also identified being illiterate as independent predictor for poor social health quality of life [17].

Environment plays a major role in determining health status. In the current finding environmental domain score was the second lowest score among all six domains.

The current study found that males had higher quality of life than their female counterparts (Table 2).

Studies in USA [24], India [28], and Vietnam [20] reported that men had higher quality of life in environmental domain. The finding that men reported higher scores on environmental domain might not be unanticipated because higher proportion of males were literates and employed and because of the fact that the domain evaluates factors such as freedom, the nature of working environment, financial resources, and participation and opportunities for leisure activities and males are expected as their social role is primary as being head of household and having decision maker power; men's have better access to the above resources as compared to women.

Spiritual/religious/personal belief quality of life had the highest score of all domains with mean ± SD = (17.0 ± 2.74 males, 16.41 ± 3.09 females) (Table 2). In contrast to study in India, our finding indicated that males had statistically significant higher score than females. The possible explanation for this disparity could be the cultural difference between the study in India [28] and the current study area. But the explanation for scoring high quality of life than the other domains could be that people tend to be spiritual and religious when confronted with issues that are beyond them; they engage in spiritual and religious reflections, treasuring the gifts in their lives, accepting and surrendering to the approach of their death, and facing the part they may have played in their own demise [29]. This could account for the observed high quality of life scores in the domain spiritual/religious/personal belief domain.

The independent predictors of spiritual/religious/personal belief domain were family size abd WHO staging for females and adherence for ART and perceived stigma for male counterparts (Table 4).

## 6. Strength and Limitation of the Study

Use of culturally adapted and validated quality of life questionnaire and permitting equal opportunity for selecting clients from all ART sites in Mekelle town were strengths of this study. The present study had some limitations that should be acknowledged. The instrument used for assessing HRQOL for this study and the study used for calculating the current sample size are not similar. Hence, information bias could have been introduced.

The respondents were ones who were actively seeking routine medical care. There are also people who do not come to the institution from the communities; therefore, generalizability is only for those who are on follow-up. Another limitation is that there could have occurred interviewer and respondent biases even if the instrument was preuse-tested. Use of self-reported measurements in adherence may cause overestimation. One more concern is that social desirability bias may have occurred. Lastly, though variables such as alcohol use, smoking, and chewing khat have been assessed, they were not controlled due to small frequencies observed.

## 7. Conclusion

This study revealed that there is a statistically significant gender difference (*P* < 0.05) in HRQOL among PLHIV on HAART. Females had low score in all HRQOL domains when compared to the male counterparts in the domains physical, psychological, level of independence, environmental, spiritual/religious/personal belief, and two general items: overall quality of life and general health perception. High perceived stigma was strongly associated with poor psychological quality of life domain among both female and male groups with [AOR = 2.89(1.69,4.96)] and [AOR = 2.5(1.4,4.4)], respectively. The independent predictors of poor physical health quality of life in both female and male groups were literacy and psychosocial support outside family members. Perceived stigma was common predictor of poor psychological health for both genders. Being illiterate and low monthly income were common predictors for poor social health quality of life domain in both genders.

Thus, to achieve global commitments including universal access to HIV/AIDS prevention, care, and treatment and Millennium Development Goals 3 and 6, there is need to measure and monitor clinical care of people living with HIV on HAART through the QOL assessment tools. Hence, National AIDS program and policy makers at different levels should realize the importance of measuring quality of life as part of the services to be provided to PLHIV. As women are most economically, culturally, and socially disadvantaged and lack equal treatment acceptance and empowerment, gender sensitive approaches should be enhanced in treatment, care, and support in dealing with PLHIV.

Different stakeholders including the PLHIV associations, religion based organizations, and other stakeholders that work in the ARV scale-up program should focus on care strategies in the area of psychological health and spiritual and environmental health directed towards PLHIV on HAART, especially women to improve their quality of life. Continued efforts are needed to reduce the stigmatizing attitudes and behavior toward PLHIV in the general population and improve support networks for PLHIV, particularly for females as they perceived stigma highly which might be barrier to seeking necessary health care and support. Health care providers should focus on counseling and providing continued education to families, friends, and relatives, to augment quality of life of PLHIV besides giving psychological support to play major role for PLHIV on HAART especially women to alleviate the burdens of HIV/AIDS related physical limitation. A large scale HRQOL research (preferably cohort) might be necessary in order to examine gender difference and address the problems of PLHIV on HAART as well as to identify changes in domain scores over time in response to treatment regarding alcohol use, smoking, and khat chewing as they were not controlled due to small frequency.

## Figures and Tables

**Table 1:** Sociodemographic characteristics, psychosocial variables, and clinical parameters of people living with HIV, in public health institutions, Mekelle town, 2012.

Sociodemographics	Gender	
	Female number (%)	Male number (%)
mean (±SD) age	35.46 (±8.03)	39.75 (±7.85)
Marital status		
Single	13 (5.2)	30 (12.3)
Divorced/separated	86 (34.4)	48 (19.7)
Married	80 (32.0)	144 (59.0)
Widowed	71 (28.4 )	22 (9.0)
Employment status		
Employed	178 (71.2)	232 (95.1)
Unemployed	72 (28.8)	12 (4.9)
Monthly income^✦^		
Below average (<83.75$)	236 (94.4)	207 (84.8)
Average and above (≥83.75$)	14 (5.6)	37 (15.2)
Religion		
Orthodox	234 (93.6)	231 (94.7)
Muslim, Protestant	16 (6.4)	13 (5.3)
Educational status		
Illiterate	81 (32.4)	30 (12.3)
Literate	169 (67.6)	214 (87.7)
Psychosocial variables		
Psychosocial support from family members		
Yes	79 (31.6)	88 (36.1)
No	171 (68.4)	156 (63.9)
Psychosocial support outside family		
Yes	179 (71.6)	164 (67.2)
No	71 (28.4)	80 (32.8)
Perceived stigma		
Low	118 (47.2)	140 (57.4)
High	132 (52.8)	104 (42.6)
Clinical parameters [extracted from client records]		
CD4 count at start of ART		
Median (IQR)^†^	163 (95–240)	137 (66–215.5)
CD4 count at start of ART		
<200	151 (63.2)	164 (70.4)
≥200	88 (36.8)	69 (29.6)
Most recent CD4 count		
Median (IQR)^†^	366 (250–523)	296 (205–441)
<200	37 (15.2)	56 (24.2)
≥200	206 (84.8)	175 (75.8)
Months on ART		
Mean (±SD)	43.57 ± 24.08	45.90 ± 24.16
Adherence to doses of ARV		
≥95% adherent	231 (92.4)	219 (89.8)
<95% adherent	19 (7.6)	25 (10.2)
Recent WHO clinical stage		
Stage I	52 (20.8)	31 (12.7)
Stage II	46 (18.4)	33 (13.5)
Stage III	35 (14.0)	51 (20.9)
Stage IV	117 (46.8)	129 (52.9)

^†^Median and IQR are reported due to nonnormal distribution, 1 USD = 17 ETB, and ^✦^the cutoff point is based on World Bank 2010 report.

**Table 2:** Gender difference in mean score of health related QOL domains of PLHIV on HAART in public health institutions, Mekelle town, Ethiopia, 2012.

Domains	*t*-test			
	Mean ± SD Female (*n* = 250)	Mean ± SD Male (*n* = 244)	*t*	*P* value^a^
Physical	15.90 ± 2.95	16.44 ± 2.76	−2.08	0.037^∗∗^
Psychological	15.54 ± 2.44	16.06 ± 2.47	−2.35	0.01^∗^
Level of independence	14.01 ± 2.86	14.60 ± 2.69	−2.37	0.01^∗^
Social relationships	13.14 ± 2.93	13.50 ± 2.71	−1.43	0.15
Environment	14.08 ± 2.43	14.51 ± 2.41	−1.98	0.04^∗^
Spiritual/personal belief	16.41 ± 3.09	17.03 ± 2.74	−2.37	0.018^∗∗^
Overall HRQOL	3.96 ± 0.91	4.11 ± 0.85	−1.94	0.05^∗^
General health perception	4.08 ± 3.52	4.27 ± 0.79	−2.53	0.01^∗^

*P* ^a^ = *P* values of independent *t* test, ^*^ *P* < 0.05, and ^**^ *P* < 0.001.

**Table 3:** Independent predictors of poor HRQOL domains among female PLHIV on HAART in public health institutions, Mekelle, Ethiopia, 2012 [*N* = 250].

Variables		PH AOR (95% CI)	Psy AOR (95% CI)	Ind AOR (95% CI)	Soc AOR (95% CI)	Env AOR (95% CI)	Spir AOR (95% CI)
Psychosocial support outside family	Yes	1			1		
	No	2.6 (1.4, 4.9)^∗^			2.8 (1.3, 6.2)^∗^		

Recent WHO stage	Stage I	0.69 (0.32, 1.48)					2.2 (1.1, 4.4)^∗^
	Stage II	0.56 (0.26, 1.2)					3.2 (1.6, 6.7)^∗^
	Stage III	2.0 (0.85, 4.98)					2.4 (1.1, 5.4)^∗^
	Stage IV	1					1

Educational status	Illiterate	1.86 (1.03, 3.35)^∗^			2.4 (1.2, 4.9)^∗^		
	literate	1			1		

Monthly income^✦^	Below average (<83.75$)	1			1	1	
	Average and above (≥83.75$)	0.27 (0.07, 0.99)^∗^			0.15 (0.03, 0.65)^∗^	0.15 (0.03, 0.70)^∗^	

Residence	Urban		1	1		1	
	Rural		6.5 (1.37, 30.66)^∗^	4.1 (1.0, 15.8)^∗^		4.1 (1.15, 14.9)^∗^	

Psychosocial support from family/friends	Yes		1		1		
	No		2.05 (1.15, 3.66)^∗^		2.1 (1.07, 4.38)^∗^		

Perceived stigma	Low		1				
	High		2.89 (1.69, 4.96)^∗^				

Months on ART	<36 months	0.48 (0.26, 0.88)^∗^		0.52 (0.29, 0.8)	0.48 (0.2, 0.92)^∗^		
	≥36 months	1		1	1		

Age category	15–24				4.7 (1.1, 20.3)^∗^		
	25–34				2.7 (1.3, 5.2)^∗^		
	35–44				1		
	≥45				0.48 (0.1, 1.1)		

Time since diagnosis	<36 months					0.35 (0.19, 0.61)^∗^	
	≥36 months					1	

Family size	≤2						2.1 (1.16, 3.72)^∗^
	≥3						1

Recent side effect	Yes			2.5 (1.01, 6.25)^∗^			

	No			1			

AOR = adjusted odds ratio, CI = confidence interval, ^*^ *P* < 0.05, ^**^ *P* < 0.001, 1 = reference category, PH = physical health, Psy = psychological health, Soc = social relationship, Env = environment, Ind = level of independence, Spir = spiritual health, 1 USD = 17 ETB, and ^✦^the cutoff point is based on World Bank 2010 report.

The Hosmer-Lemeshow goodness-of-fit test statistic is greater than 0.05 for all the models.

**Table 4:** Independent predictors of Poor HRQOL domains among male PLHIV on HAART in public health institutions, Mekelle, Ethiopia, 2012 [*N* = 244].

Variables		PH AOR (95% CI)	Psy AOR (95% CI)	Ind AOR (95% CI)	Soc AOR (95% CI)	Env AOR (95% CI)	Spir AOR (95% CI)
Psychosocial support outside family	Yes	1					
	No	2.4 (1.29, 4.46)^∗^					

WHO stage recent	Stage I	0.15 (0.05, 0.44)^∗^	0.24 (0.09, 0.63)^∗^	0.12 (0.04, 0.34)^∗∗^		0.31 (0.13, 0.76)^∗^	
	Stage II	0. 50 (0.21, 1.18)	0.14 (0.04, 0.4)^∗^	0.20 (0.08, 0.51)^∗∗^		0.16 (0.06, 0.46)^∗∗^	
	Stage III	1.18 (0.6, 2.3)	1.1 (0.55, 2.2)	0.42 (0.21, 0.85)^∗∗^		0.86 (0.44, 1.69)	
	Stage IV	1	1	1		1	

Educational status	Illiterate	3.26 (1.35, 7.85)^∗^			3.1 (1.2, 7.9)^∗^		
	literate	1			1		

Monthly income^✦^	Below average (<83.75$)			1	1		
	Average and above (≥83.75$)			0.32 (0.14, 0.77)^∗∗^	0.20 (0.08, 0.46)^∗∗^		

Perceived stigma	Low	1	1	1			1
	High	1.88 (1.06, 3.25)^∗^	2.5 (1.44, 4.44)^∗^	2.90 (1.6, 5.41)^∗∗^			3.58 (2.03, 6.32)^∗∗^

Marital status	Married				1		
	Single/widowed/ divorced/ separated				1.8 (1.04, 3.19)^∗^		

Time since diagnosis	<36 months				0.54 (0.30, 0.98)^∗^		
	≥36 months				1		

Family size	≤2					1.9 (1.03, 3.69)^∗^	
	≥3					1	

Adherence status	Yes					1	1
	No					2.5 (1.01, 6.26)^∗^	2.62 (1.16, 6.25)^∗^

AOR = adjusted odds ratio, CI = confidence interval, ^*^ *P* < 0.05, ^**^ *P* < 0.001, 1 = reference category, PH = physical health, Psy = psychological health, Soc = Social relationship, Env = Environment, Ind = level of independence, Spir = Spiritual health, 1 USD = 17 ETB, and ^✦^the cutoff point is based on World Bank 2010 report.

The Hosmer-Lemeshow goodness-of-fit test statistic is greater than 0.05 for all the models.
